# Metabolic features in chronic HCV infected patients

**DOI:** 10.1186/1471-2334-13-S1-O31

**Published:** 2013-12-16

**Authors:** Mihaela Andreea Rădulescu, Victoria Aramă, Daniela Ioana Munteanu, Cristina Popescu, Raluca Mihaela Năstase, Violeta Molagic, Anca-Ruxandra Negru, Irina Lăpădat, Viorica Poghirc, Sorin Ștefan Aramă, Adrian Streinu-Cercel

**Affiliations:** 1 National Institute for Infectious Diseases “Prof. Dr. Matei Balş”, Bucharest, Romania; 2 Carol Davila University of Medicine and Pharmacy, Bucharest, Romania; 3 Dr. Ioan Cantacuzino Clinical Hospital, Bucharest, Romania

## Background

In recent years a large amount of literature data emphasizes the interrelationship between pathogenic mechanisms of chronic hepatitis C virus (HCV) infection and lipid and glucid metabolism. In this study we aimed to characterize lipid and glucidic metabolic patterns in chronically HCV-infected patients and to evaluate the role of HCV on cardiovascular risk (CVR).

## Methods

We conducted a cross-sectional analysis on chronic HCV-infected adult patients, monitored at the National Institute of Infectious Diseases “Prof. Dr. Matei Balş”. Patients with diabetes mellitus, chronic alcohol consumption, other chronic liver diseases, HBV or HIV co-infections were excluded. We recorded demographic data, HCV infection history, personal and family history of CVR factors. We measured height and weight for body mass index, waist to hip ratio, blood pressure and we assessed the 10 years CVR using Framingham score. Blood samples were tested for lipid profile, serum glucose, glycosylated hemoglobin (HbA_1c_), liver enzymes, and viral load (VL). Liver histology was assessed by Fibromax (Biopredictive).

## Results

Seventy-six patients with a median age of 51years (IQR 44.25-58.0) were included. Sex ratio was F:M=1.53. Median VL was 118500 IU/mL (IQR 0-600951). Twenty-five percent (19/76) of the patients had no fibrosis (F0), 51.3% (39/76) had hepatitis (F1-2), 6.5% (5/76) had a fibrosis score equivalent to transition to cirrhosis (F3), and 17.1% (13/76) had cirrhosis (F4). Mean serum cholesterol, LDL and triglycerides were 187 mg/dL (IQR 166-220), 119 mg/dL (IQR 93-147) and respectively 94 mg/dL (IQR 69.25-132.0). Patients with no fibrosis were more frequently younger and females (p=0.000, respectively 0.015), had higher cholesterol (p=0.014) and LDL levels (p=0.009) and lower VL (p=0.017) and CVR according to Framingham score (p=0.000). Patients with cirrhosis were more frequently males (p=0.033), and had higher viral load (p=0.011) and serum glucose (p=0.027). Fibrosis score correlated to age (p=0.000), VL (p=0.001), higher CVR (p=0.001), low LDL (p=0.034), high glucose (p=0.031). The VL correlated to HDL (p=0.023) and with lower HbA_1c_ levels (p=0.022).

## Conclusions

In patients with chronic HCV infection although high fibrosis correlates to better lipid profiles, it also correlates to higher cardiovascular risk.

